# The combined impacts of wheat spatial position and phenology on cereal aphid abundance

**DOI:** 10.7717/peerj.9142

**Published:** 2020-05-26

**Authors:** Zhaniya S. Batyrshina, Alon Cna’ani, Tamir Rozenberg, Merav Seifan, Vered Tzin

**Affiliations:** 1 French Associates Institute for Agriculture and Biotechnology of Drylands, Jacob Blaustein Institutes for Desert Research, Ben-Gurion University of the Negev, Sede Boqer campus, Israel; 2 Mitrani Department of Desert Ecology, Swiss Institute for Dryland Environmental and Energy Research, Jacob Blaustein Institutes for Desert Research, Ben-Gurion University of the Negev, Sede Boqer campus, Israel

**Keywords:** Wheat, Pest, Insect, Plant diversity, Aphid infestation, Plant developmental stages, Agroecosystem, Flavonoid, Specialized metabolites, Neighboring resources

## Abstract

**Background:**

Wheat is a staple crop that suffers from massive yield losses caused by cereal aphids. Many factors can determine the abundance of cereal aphids and the damage they cause to plants; among them are the plant’s genetic background, as well as environmental conditions such as spatial position within the plot, the composition and the distance from neighboring vegetation. Although the effects of these factors have been under scrutiny for many years, the combined effect of both factors on aphid populations is not fully understood. The goal of this study was to examine the collective impact of genotype and environment on wheat phenology (developmental stages), chemical diversity (metabolites), and insect susceptibility, as manifested by cereal aphid abundance.

**Methods:**

To determine the influence of plant genotype on the metrics mentioned above, we measured the phenology, chemical profile, and aphid abundance of four wheat genotypes, including the tetraploid wild emmer (*Triticum turgidum* ssp. dicoccoides cv. Zavitan), tetraploid durum (*Triticum turgidum* ssp. durum cv. Svevo), and two hexaploid spring bread (*Triticum aestivum*), ‘Rotem’ and ‘Chinese Spring’. These genotypes are referred to as “focal” plants. To evaluate the impact of the environment, we scored the distance of each focal plant (spatial position) from two neighboring vegetation types: (i) natural resource and (ii) monoculture wheat resource.

**Results:**

The results demonstrated that the wild emmer wheat was the most aphid-resistant, while the bread wheat Rotem was most aphid-susceptible. Aphids were more abundant in plants that matured early. The spatial position analysis demonstrated that aphids were more abundant in focal plants located closer to the margin monoculture wheat resource rather than to the natural resource, suggesting a resource concentration effect. The analysis of metabolic diversity showed that the levels of three specialized metabolites from the flavonoid class, differed between the wheat genotypes and some minor changes in central metabolites were shown as well. Altogether, these results demonstrate a combined effect of genetic background and spatial position on wheat phenology and aphid abundance on plants. This exposes the potential role of the marginal vegetation environment in shaping the insect population of desirable crops. These findings highlight the importance of maintaining plant intra-specific variation in the agriculture system because of its potential applications in reducing pest density.

## Introduction

Wheat is a staple crop that provides 20% of the world population’s caloric and protein intake (FAOSTAT, 2014; Shewry & Hey, 2015). With the world’s growing population, the demand for food is predicted to increase 40% by the year 2050, triggering an urgent need to increase crop yield (Hunter et al., 2017). One of the main reasons for crop loss is pest damage, with an average 15% reduction in grain quality and yield (Lee et al., 1981; Deutsch et al., 2018). The most dominant group of wheat pests is aphids (Hemiptera: Aphididae) (Vickerman & Wratten, 1979; Rabbinge et al., 1981). Aphids affect plant production by causing a reduction in nutrients, diminished photosynthesis efficiency, reallocation of source-sink relationships (Rabbinge et al., 1981; Bing et al., 1991), and transmission of plant viruses (Fereres et al., 1989; Nault, 1997). Aphids are phloem-feeding insects that use their mouthparts to penetrate host tissue causing a minimal amount of damage (Douglas, 2003). Once aphids find a suitable feeding site, they can ingest phloem sap for hours or even days (Nalam, Louis & Shah, 2019). Among the most economically important aphids to the Gramineae family plant species are the bird cherry-oat aphid (*Rhopalosiphum padi* L.), English grain aphid (*Sitobion avenae* Fabricius), and greenbug (*Schizaphis graminum*), which are commonly referred to as “cereal aphids” (Blackman & Eastop, 2000; Parry, 2013).

The relationship between host plants and insect abundance depends on internal factors, such as genetic background, as well as external environmental factors including host plant density and diversity, vegetation patch size and the level of landscape complexity (Tahvanainen & Root, 1972; Root, 1973; Finch & Collier, 2000; Rhainds & English-Loeb, 2003; Joshi et al., 2004; Otway, Hector & Lawton, 2005). An example of an internal factor effect is the variation in wheat genetic background as related to determining the response to aphids. The ancient diploid wheat *Triticum monococcum* genotypes were shown to be entirely or partially resistant to cereal aphids while the hexaploid (*Triticum aestivum*) genotypes were more susceptible (Spiller & Llewellyn, 1986; Migui & Lamb, 2003). An example of an external factor is the effect of plant diversity gradients on insect population herbivory and predator abundance, which ranges from positive impacts (Mulder et al., 1999; Dinnage, 2013; Loranger et al., 2014) to negative impacts (Unsicker et al., 2006), as well as no significant effects. These variations were shown for both herbivore and predator abundance and richness of the insect species (Scherber et al., 2006; Claflin et al., 2017). A meta-analysis approach based on 21 studies conducted in 1984–1994 on crop diversity on herbivorous insects concluded that crop diversity caused a mild reduction in insect populations (Tonhasca & Byrne, 1994). However, in a more recent meta-analysis based on 46 studies, no significant response of herbivorous insect abundance to landscape complexity was found, but there was a strong positive response to landscape complexity for natural enemies (Chaplin-Kramer et al., 2011). Since crop-diversifying pest management strategies are debatable (Médiène et al., 2011), further studies are required to better understand landscape effects and spatial organization impacts on pest abundance on specific plant species.

To cope with insect damage, plants synthesize a large array of compounds, including central, and specialized defensive metabolites (Kessler & Halitschke, 2007; Schmidt, Schurr & Röse, 2009). While many of these compounds are produced constitutively, regardless of insect attack, others are normally present at basal levels and become more abundant (induced) in response to insect feeding (Bennett & Wallsgrove, 1994; Boughton, Hoover & Felton, 2005). In response to aphid infestation, wild and domesticated tetraploid wheat (*Triticum turgidum*) modify the production of phytohormones and defense metabolites from the benzoxazinoid class (Chandrasekhar et al., 2018; Shavit et al., 2018). Additionally, in response to *R. padi* aphid damage, the diploid *Triticum monococcum* genotypes modify the levels of several central metabolites, including amino-, organic-, and nucleic acids (Greenslade et al., 2016). Silencing of two genes from the terpenoid pathway, *S-linalool synthase* and the *(E)-*β*-caryophyllene synthase* in rice (*Oryza sativa*) affect volatile emission, which resulted in differences in insect communities in field conditions, highlighting the importance of chemical diversity in controlling pest populations (Xiao et al., 2012). Metabolic diversity allows plant resistance to different herbivores, and transmission of information to other organisms such as neighboring plants, predators and parasitoids (Baldwin et al., 2006; Kessler & Kalske, 2018).

Plant phenology (developmental stages) is known to affect insect population dynamics. For example, *R. padi* aphids change their feeding site and reproduction rates according to the developmental stage of either barley (*Hordeum vulgare*), oat (*Avena sativa*), or wheat (Leather & Dixon, 1981). It was previously reported that aphids prefer to feed on young seedlings of winter-wheat during the spring, and mature flowering plants of spring wheat during the summer (Migui & Lamb, 2003). In response to insect attack, plants can modify their phenology in order to delay their growth or escape by an early transition into the reproductive stage (Mitchell et al., 2016). Furthermore, western corn rootworm (*Diabrotica virgifera*) herbivore tolerance involves delayed over-compensatory root regrowth in maize (Mitchell et al., 2016), which might improve insect tolerance by postponing plant development until the attacker moves away from the plant (Tiffin, 2000). Overall, plant metabolism and phenology are reprogramed under biotic and abiotic stresses (Kessler & Halitschke, 2007).

The spatial position of the focal plant relative to the neighboring plants (outside the plot) and the other focal plants (inside the plot) influences insect abundance, which affects resistance or susceptibility (Kos et al., 2015; Cohen & Crowder, 2017). It was previously reported that the spatial plant position of potato (*Solanum tuberosum*, the focal plant), is affected by the presence of the marginal neighboring plants onion (*Allium cepa*) and garlic (*Allium sativum*). This study implied that volatile compounds that were emitted from neighboring onion and garlic plants reduced the abundance of green peach aphid (*Myzus persicae*) on potato plants (Ninkovic et al., 2013). A previous study reported that plant diversity in agroecosystems is shown to enhance insect natural enemy communities, thereby leading to a reduction in herbivore populations (Letourneau et al., 2011). Diversity can be enhanced in agroecosystems by multiple cover crops, agroforestry, crop and livestock mixtures, intercropping (diversity within the focal plant plots), or by the surrounding landscapes and marginal vegetation (bordering the focal plant plots) (Altieri & Nicholls, 2019). These methods are occasionally used as sustainable approaches to promote biological control by increasing the presence of natural enemies, thus reducing synthetic insecticide applications (Ninkovic et al., 2013; Tulli, Carmona & Vincini, 2013; Lopes et al., 2016). The focal plant’s density might also affect herbivore. For example, in the common ragwort plant (*Senecio jacobaea*), young caterpillars of cinnabar moth (*Tyria jacobaeae*) are more prevalent when hosts are closely spaced, while older caterpillars prevail on more widely spaced plants (Kunin, 1999). In the case of specialist herbivores, the plant density can cause a resource concentration when the host plants grow in high-density patches and low-diversity mixtures (Altieri, 1995; Rhainds & English-Loeb, 2003), or resource dilution depends on the population of natural enemies (Otway, Hector & Lawton, 2005). Overall, the spatial position and density are crucial parameters that affect insect resistance and therefore, in some cases, exploited to improve agroecosystems by reducing insect pest densities and pesticide applications (Ricci et al., 2009; A’Bear, Johnson & Jones, 2014; Alignier et al., 2014).

Although many studies have previously reported on the impact of wheat genotypes on various insects in the field, the combined impacts of spatial position, genetic background, and phenology on aphids are not adequately addressed. Here, we aim to elucidate the impact of these multiple factors on insect abundance, with a focus on the staple crop wheat and its major pest, cereal aphids. As a model for intra-specific phenological and metabolic diversity, we used four wheat genotypes that differ in their polyploidy levels and domestication history (Pont et al., 2019) as focal plants. This includes the following genotypes: (i) tetraploid wild emmer wheat (*Triticum turgidum* ssp. dicoccoides), named ‘Zavitan’, (ii) cultivated tetraploid durum wheat (*Triticum turgidum* ssp. durum) named ‘Svevo’, and (iii–iv) two hexaploid spring bread wheat (*Triticum aestivum*), ‘Rotem’ and ‘Chinese Spring’. We tested whether aphid abundance is correlated to plant genotype, phenology, and combined effects. The focal wheat plants were placed between two margin vegetations: wheat resource, and natural resource growing adjacent to the focal plants. We then tested whether insect abundance is influenced by spatial position relative to resource vegetation composition and combined effects of the genetic background.

## Materials and Methods

### Plants and experimental design

The experiment was carried out on the Sede Boqer campus in southern Israel (latitude: 30.87417, longitude: 34.79639), during winter and the following spring (sown on mid-December 2017, and harvested by the end of April 2018). The field plot was planned in a north–south oriented strip according to the available space on site (Fig. S1). Four wheat genotypes were chosen: wild emmer wheat ‘Zavitan’ (*Triticum turgidum* ssp. dicoccoides), durum wheat ‘Svevo’ (*Triticum turgidum* ssp. durum), and two hexaploid bread wheat (*Triticum aestivum*) genotypes ‘Rotem’ (Agridera Seed & Agriculture Ltd., Gedera, Israel) and ‘Chinese Spring’, which is widely used as a standard for wheat cytogenetic research (Sears & Miller, 1985). Seed material from the Svevo, Zavitan, and Chinese Spring genotypes was characterized and provided by Prof. Assaf Distelfeld (University of Haifa, Israel). Seeds were sown during mid-December 2017 at eight cm apart, approximately three cm deep in the soil, at a total of 16 seeds per row. The experiment was designed in three blocks, each including the four genotypes in a different arrangement. Seeds of each genotype were sown in two rows of each block (see Fig. S1 for field setup). The experimental block was composed of eight rows at four units long and two units wide with 1.9 m between block #1 and block #2, and 4.0 m between block #2 and block #3, respectively (Fig. S1; Table S1). Before starting the experiment, the soil was prepared by using a rotary tiller where the focal plants and wheat resources were sown. In total, 96 seeds were sown from each genotype, and the seed germination and maturation resulted in the following numbers of plants: 74 Svevo, 61 Zavitan, 65 Rotem, and 67 Chinese Spring. Wheat plants require a nitrogen source, which is necessary for growth and grain filling (Imran et al., 2019), thus, fertilizers were applied once to enhance soil nitrogen content and organic compost at the beginning of the season. The field was fertilized with a nitrogen source (urea 46%; ICL Ltd., Tel Aviv-Yafo, Israel) at 150 kg per hectare, a superphosphate mix (Ca[H_2_PO_4_]_2_+CaSO_4_; ICL Ltd., Tel Aviv-Yafo, Israel), at 100 kg per hectare, and a Garden Mix (Hagarin Ltd., Karmiel, Israel) at 10 m^3^ per hectare. An irrigation system using sprinklers coupled with rainfall (average 100 mm per year) provided water once a week or according to demand. During the growing season, we regularly weeded experimental blocks and the wheat resource while the vegetation of the natural resource reminded untreated.

### Margin vegetations

The three blocks of four focal wheat genotypes were adjacent to two different vegetation types: (i) wheat resource, cultivated monoculture hexaploid bread (Agridera Seeds and Agriculture Ltd., Gedera, Israel) wheat on the east side (approximate height of the plants was 70–85 cm), and (ii) natural resource on the west side (approximate height of the plants was 10–60 cm). To identify plant diversity within natural resources, we used the Flora of Israel Online database (Danin & Fragman-Sapir, 2016; Table S2).

### Wheat phenology

To evaluate the plant phenology, the wheat plants were scored according to the Feekes growth scale categories: (i) tillering stages 1–5, (ii) stem extension stages 6–10, (iii) heading stages 10.1–10.5 and (iv) ripening stage 11 (Large, 1954). Since the plants were approximately three months old, they were in the final stages of the Feekes scale at 8–10.5. Overall, plants were in advanced mature stages, divided into two subcategories: (i) stem extension including (8) last leaf just visible, (9) ligule of last leaf visible, and (10) booting; and (ii) heading, including (10.1) the first ear just visible, and (10.5) flowering.

### Aphid abundance on wheat plants

For evaluating aphid abundance, individual focal wheat plants of the four genotypes were scored for aphids in all three blocks. The total number of aphids were counted on the entire aboveground vegetative tissue; aphids were mostly found on flag leaves and spikes. The counting was conducted twice in a 10-day interval: first on March 20th, 2018, and again on April 1st 2018. We decided to take this measurement in a 10-day interval between progeny counting due to aphid life cycle features that might differ in a season-dependent manner. Because our experiments were conducted in spring/summer, we consider that aphids reproduce parthenogenetically, and it will take 8–10 days for the new generation to mature.

### Metabolic diversity analysis using ultra-performance liquid chromatography coupled with a quadrupole time-of-flight mass spectrometer

For studying wheat metabolic profile, the flag leaves collected and aphids (if present) were removed before tissue harvesting. The tissues were sampled in alternate order of genotypes during an hour (at noon) of harvesting samples. Samples were weighed and extracted in 80% methanol, 19.9% MilliQ water (EMD Millipore Corp., Burlington, MA, USA), and 0.1% formic acid at a 1:10 (w:v) ratio. After a brief vortex, tubes were shaken for 40 min at 4 °C and centrifuged for 5 min at maximum speed. Samples were then filtered through a 0.22-μm filter plate (EMD Millipore Corp., Burlington, MA, USA) using a centrifuge at 3,000*g* for 5 min (Mijares et al., 2013). Then, 2 μl were injected onto an ultra-performance liquid chromatography (UPLC) machine coupled with a quadrupole time-of-flight mass spectrometer (qTOF-MS) instrument equipped with an ESI interface (Waters MS Technologies, Manchester, UK) using ACQUITY UPLC BEH C18 column (100 mm × 2.1 mm, 1.7 μm, Waters). The analysis was done in both negative and positive ion modes. The MS conditions were set essentially as described previously (Hochberg et al., 2013). MassLynx software (Waters) version 4.1 was used for system control and data acquisition. For metabolite identification, we used authentic standards (from commercial sources), based on retention time and fragmentation. Additionally, metabolites were also annotated based on fragmentation patterns searched against the Chemspider metabolite database (http://www.chemspider.com/) constant for retention time, fragmentation, and comparison with the data in the current scientific literature (Reshef et al., 2017). Raw data is presented in Table S3, and metabolite identification is shown in Table S4.

### Statistical analysis

For data reduction of the untargeted metabolic dataset, we conducted the partial least squares discriminant analysis (PLS-DA) plots using the MetaboAnalyst 3.0 software (Xia et al., 2009), following these parameters: missing value estimation, remove features with more than 50% and replace by a small value, and data filtering of interquartile range. Data were normalized to the median, then transformed into log scale and auto-scaled. The one-, and two-way analysis of variance (ANOVAs) of the aphid abundance, phenology, and metabolite levels, were performed using JMP13 software (SAS; www.jmp.com), followed by using Microsoft Excel for figure presentation. The effect of plant distance from the margin wheat resource, aphid observation date, and phenology on the number of aphids were tested using two repeated measures generalized linear mixed models (rmGLMM) with Poisson distribution and log link. In the first analysis, we tested for the effect of distance from the focal wheat (fixed factor) with counting dates as a repeated observation and the block (field patch) as a random factor. In the second analysis, we tested for the effect of the distance from the margin wheat resource and the phenology (developmental stage) as fixed factors, with the field patch treated as a random factor. We also tested the same statistical models on each genotype separately. In Table S1, the raw data used in the study is presented including sampling date, block number (1–3), row number ID, unit (A-natural resource; B-margin wheat resource), distance from the resource (1–8), genotype, developmental stage (categorized by Feekes scale) and aphid number.

## Results

### Aphid distribution in the four wheat genotypes

**Figure 1:**
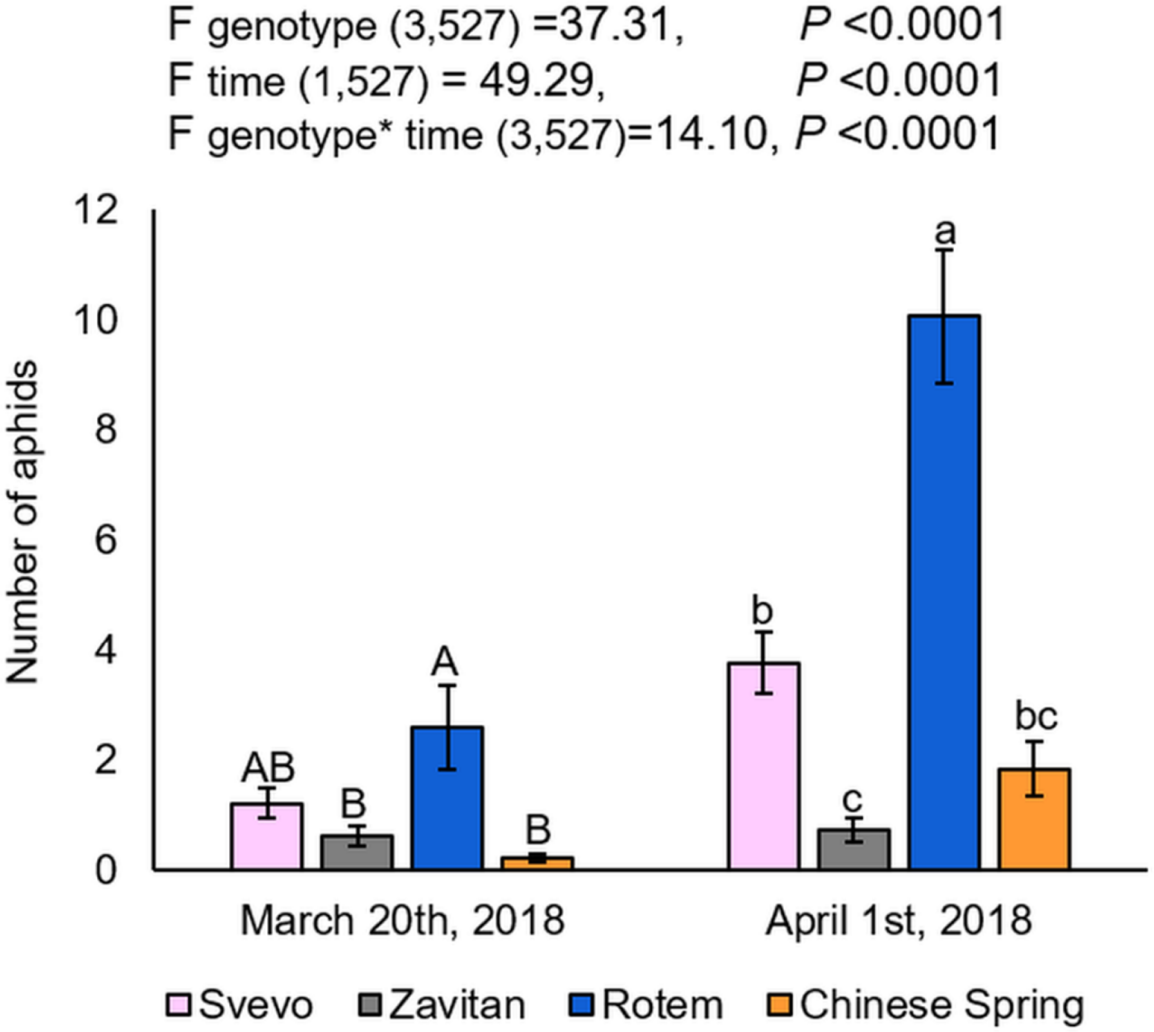
Aphid distribution in the four wheat genotypes. The number of aphids on each genotype of the focal wheat plants were counted on two dates: on March 20th and April 1st, 2018 (mean ± SE, number of replicates: Svevo = 74; Zavitan = 61; Rotem = 65; and Chinese Spring = 67). Different letters above the bars indicate significant differences between genotypes for each counting date separately (*P*-value ≤ 0.05), using the one-way ANOVA (Tukey-Kramer HSD post-hoc tests).

Overall, three cereal aphid species were identified in the field: *Sitobion avenae* (English grain aphid), *Schizaphis graminum* (greenbug), and *Rhopalosiphum padi* (bird cherry-oat aphid). The aphids were mainly found on the plants’ matured organs such as flag leaves and spikes, rather than on the juvenile vegetative tissues. The aphids’ abundance was also calculated for each block separately, which showed no differences between them (Fig. S2). The two-way ANOVA suggested a significant difference between the four wheat genotypes (*F*_3,527_ = 37.31, *P*-value < 0.0001), the counting dates (*F*_1,527_ = 49.29, *P*-value < 0.0001), and the interaction between them (*F*_3,527_ = 14.10, *P*-value < 0.0001), indicating the effects of both genotype and the time of counting on aphid abundance. As presented in Fig. 1, the number of aphids was significantly higher on Rotem relative to the other genotypes, while Svevo had a moderate number of aphids, and Zavitan and Chinese Spring had the least. The differences in aphid abundance between the genotypes were consistent on the second counting date (April 1st, 2018), but the overall aphid abundance was higher than on the first counting date. Specifically, the comparison between the two counting dates revealed a significant increase in the number of aphids on wheat plants, with the exception of Zavitan. The results suggest that Rotem is the most susceptible cultivar, whereas Zavitan plants demonstrated superior resistance.

### Phenology of the four wheat genotypes

**Figure 2:**
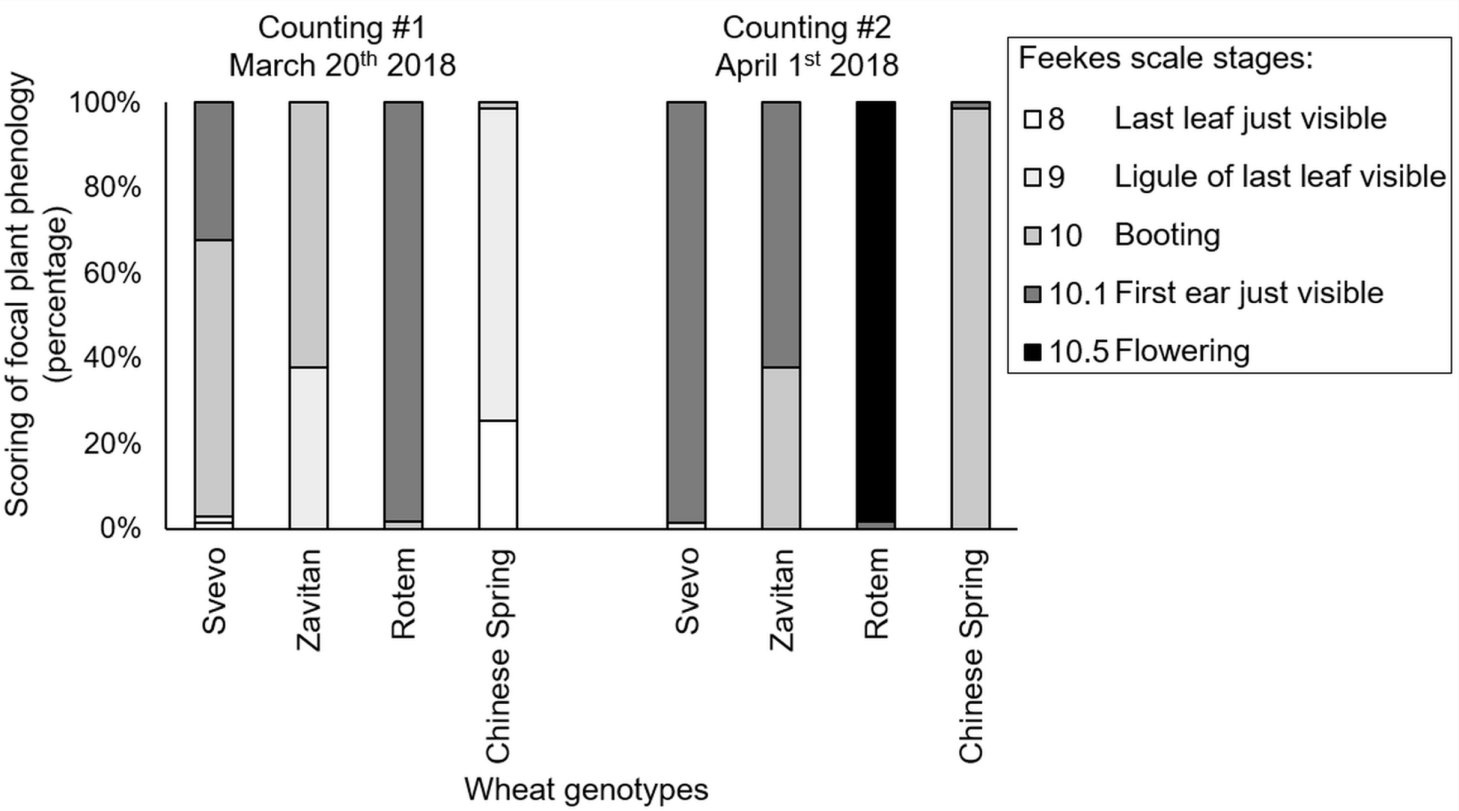
Phenology scores of four wheat genotypes. Developmental stages of the focal plants are presented as percentages (number of replicates: Svevo = 74; Zavitan = 61; Rotem = 65; and Chinese Spring = 67). Phenology evaluation was performed in 10 days interval on March 20th and April 1st, 2018 and ranked according to the Feekes scale.

We evaluated plant phenology by scoring the plants’ developmental stages according to the Feekes growth scale (Large, 1954). Overall, the two-way ANOVA suggested a significant difference in the phenology of the four wheat genotypes (*F*_3,533_ = 582.40, *P*-value < 0.0001), the counting dates (*F*_1,533_ = 296.65, *P*-value < 0.0001), and the interaction between them (*F*_3,527_ = 123.31, *P*-value < 0.0001), indicating the effect of both genotype and the time of scoring on the plant phenology. Specifically, Rotem showed significantly advanced stages of development (10.1 and 10.5), while the most lagging genotypes were Zavitan (9, 10 and 10.1), and Chinese Spring (8, 9 and 10). Svevo showed intermediate development (10 and 10.1). The developmental lag between the genotype was generally conserved on the second sampling date (Fig. 2). A correlation coefficient analysis of the aphid performance data and phenology of the two counting dates demonstrated a positive correlation between these parameters (correlation coefficient *r* value = 0.64; Fig. S3).

### Metabolic diversity between the four wheat genotypes

**Figure 3:**
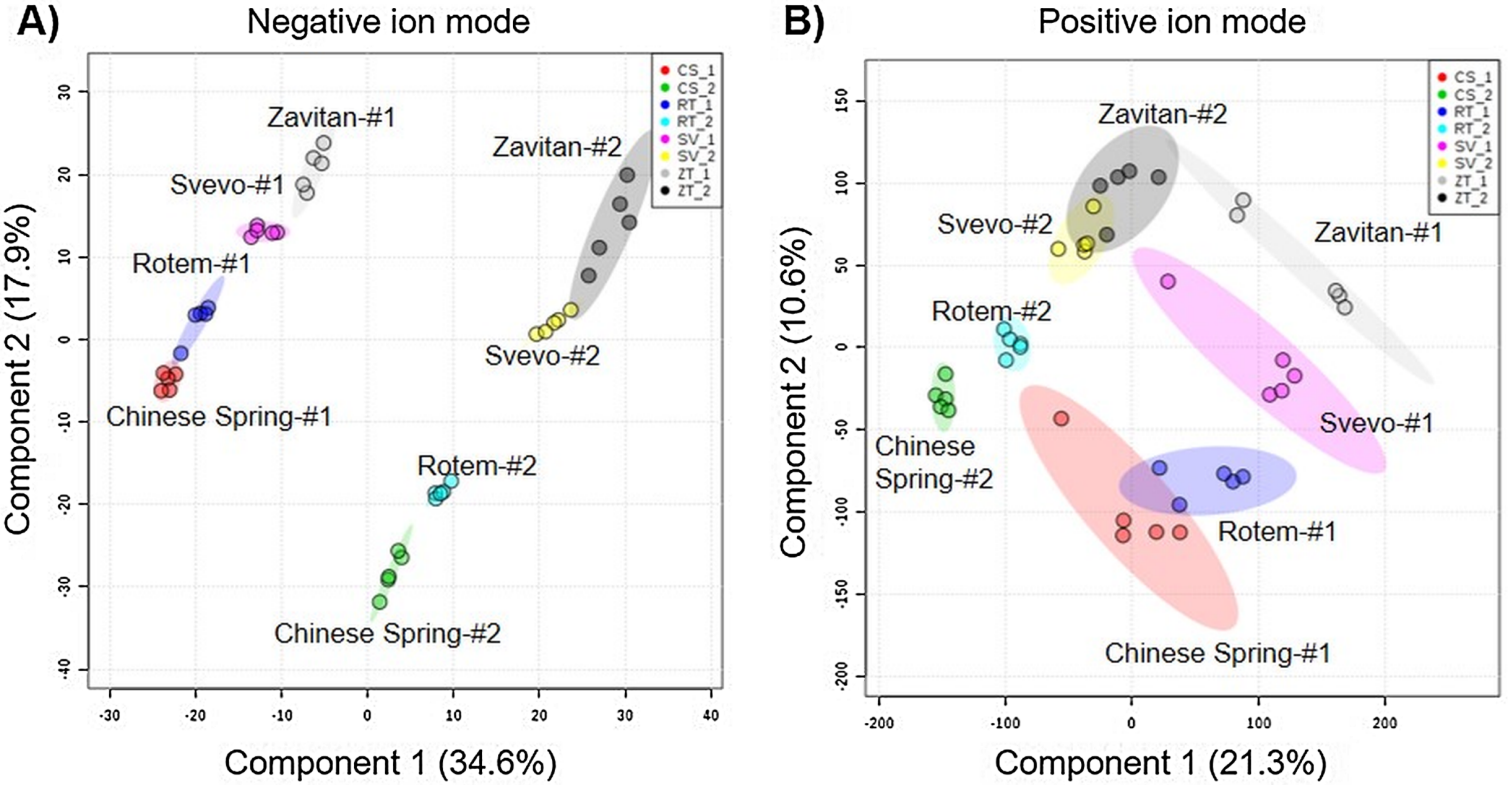
Partial least squares discriminant analysis (PLS-DA) plots of the untargeted metabolic overview of four wheat genotypes in two sampling dates obtained by the LC-MS. The PLS-DA plots comprise 2,992 features in the negative ion mode (A), and 4,981 features in the positive ion mode (B) #1 - first counting date March 25th 2018, and #2 - second counting date April 13th 2018. *n* = 5.

**Figure 4:**
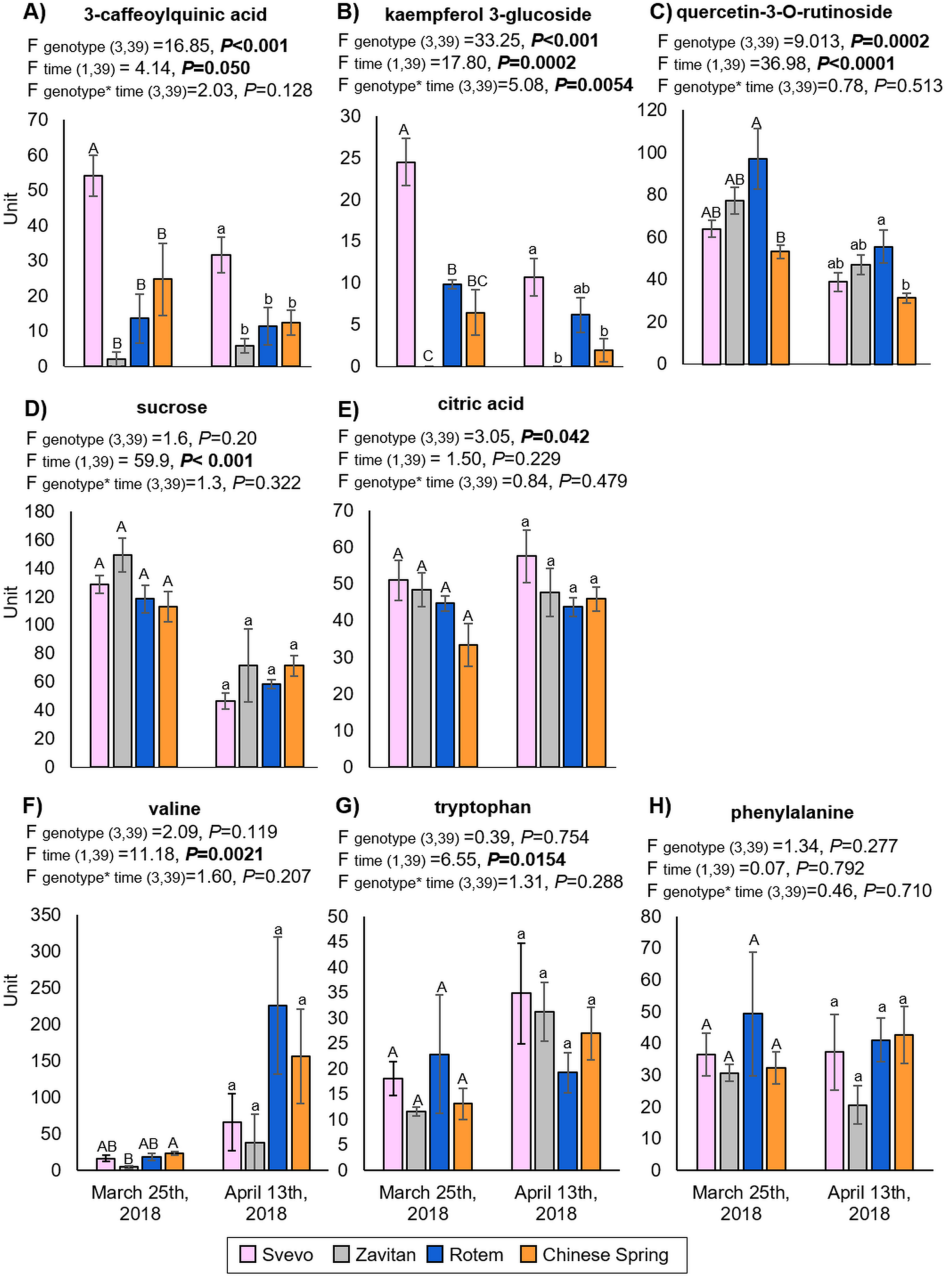
The relative levels of eight metabolites detected in the wheat leaves. (A) 3-Caffeoylquinic acid, (B) kaempferol 3-glucoside, (C) quercetin-3-O-rutinoside, (D) sucrose, (E) citric acid, (F) valine, (G) tryptophan and (H) phenylalanine. The levels are presented in units (integration for the LC-qTOF-MS), mean ± SE, *n* = 5. Different letters above the bars indicate significant differences between genotypes for each counting date separately (*P* value ≤ 0.05), using the one-way ANOVA (Tukey-Kramer HSD post-hoc tests).

We tested diversity within the plant metabolome using UPLC-qTOF-MS. In total, 2,992 features were detected in negative ion mode and 4,981 features in positive ion mode. The feature number represents features that were detected in all samples (Table S3). For data reduction, a partial least squares discriminant analysis (PLS-DA) was conducted. The percentage of the variance of components 1 and 2 in negative ion mode was equal to 52.5%, and the positive ion mode scored 31.9% (Fig. 3). The PLS-DA plot of the negative ion mode showed significant separation between the first and the second sampling date. Moreover, at the second counting date, wheat genotypes were clustered by their ploidy, indicating more similarity between the tetraploid than the hexaploid wheat (Fig. 3A). The PLS-DA plot of the positive ion mode showed that major clustering is related to the genotype but not affected by the sampling time. Zavitan samples from both sampling dates, were closer to each other and to Svevo, than to Rotem and Chinese Spring (Fig. 3B). Overall, both PLS-DA plots indicated a unique metabolic profile for each genotype, also showing some effect from the sampling time. We identified eight metabolites including three flavonoids:3-caffeoylquinic acid, quercetin-3-O-rutinoside, and kaempferol 3-glucoside, three amino acids (valine (Val), phenylalanine (Phe), and tryptophan (Trp)), a sugar (sucrose), and an organic acid (citric acid). Metabolite annotations based on retention time, fragmentation patterns and previous studies, are presented in Table S4. The relative levels of the eight compounds are represented in Fig. 4. The two-way ANOVA indicated that the levels of the three flavonoids (Figs. 4A–4C) were significantly different between the four genotypes, and between the two sampling times. The levels of sucrose, Val, and Trp (Figs. 4D, 4F and 4G) were only affected by the sampling time (reduced and increased respectively), while the level of citric acid (Fig. 4E) was only affected by the genotype. Phe (Fig. 4H) was affected by genotypes or sampling dates. Overall, the target metabolite analysis exposed metabolic changes, mainly of the three flavonoids.

### The combined impacts of wheat phenology, genotype and distance from marginal vegetation on aphid abundance

**Figure 5:**
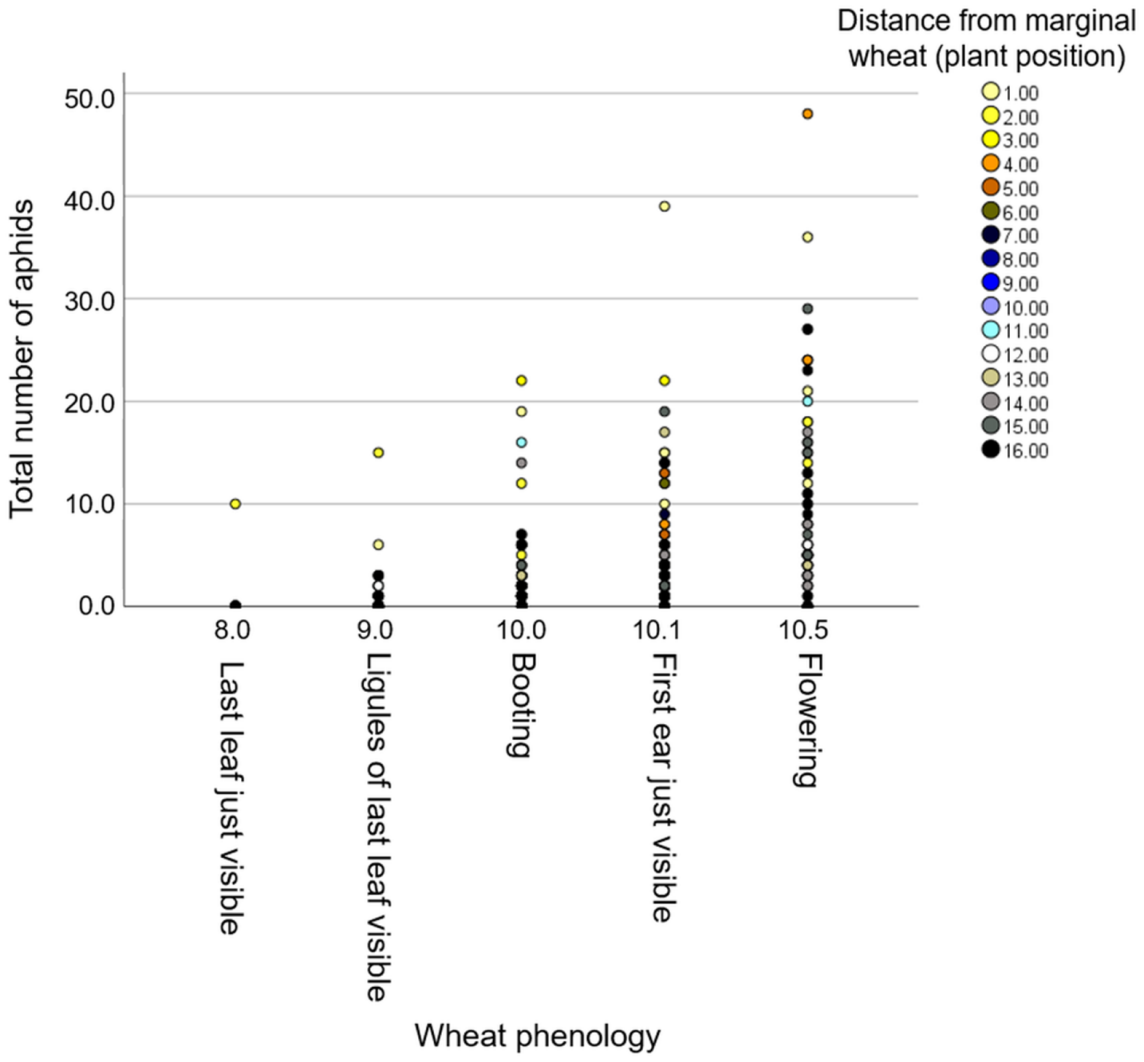
The effect of phenology on aphid number in relation to distance from the wheat resource vegetation. The positions of the plants rank from #1 (closest) to #16 (furthest) relative to the margin wheat resource.

The three wheat blocks (focal plants) were located between two neighboring vegetation types, including (i) “natural resource” and (ii) “wheat resource”, which represent wild and monoculture resource vegetations, respectively (Fig. S1). To evaluate the effect of the environment, we characterized plant diversity within the natural resource area and found seven plant families—Asteraceae, Brassicaceae, Caryophyllacaeae, Chenopodiaceae, Fabaceae, Malvaceae, and Poaceae—all common in South Israel (Table S2). We documented the positions of each individual focal wheat plant within the three blocks along the row. We first tested for the effect of the counting date and the focal position (distance from the wheat resource) on aphid abundance. The model showed significant effects from distance (*F*_1,530_ = 4.88, *P*-value = 0.028) and counting date (*F*_1,530_ = 11.39, *P*-value < 0.001), while the interaction of the two was not significant (*F*_1,530_ = 0.12, *P*-value = 0.728). The results indicate that the further the wheat plants were from the wheat resource, the fewer aphids were present on it (Fig. 5). Also, there were more total aphids on older plants (Fig. S3).

**Figure 6:**
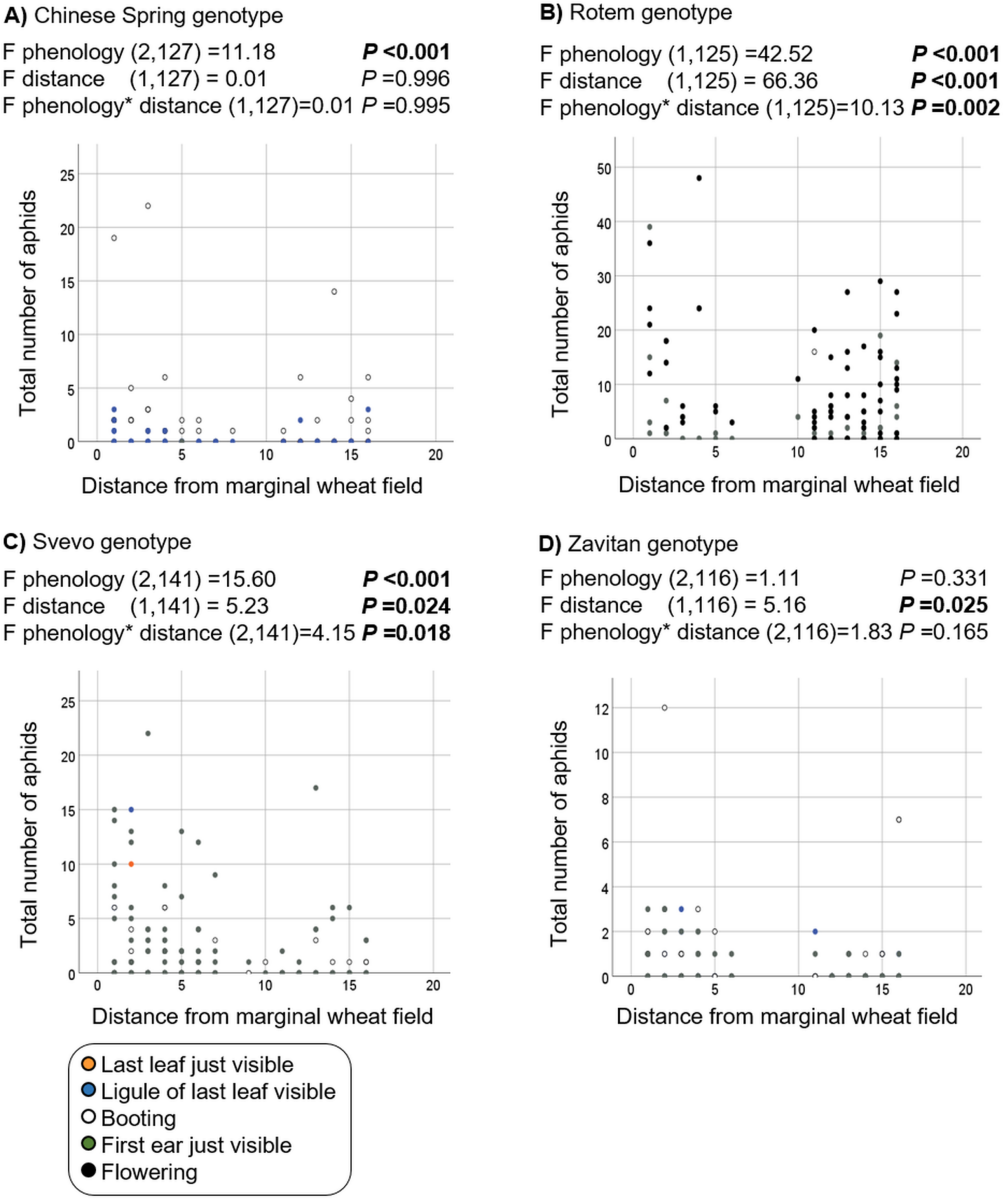
Effect of phenology and position in relation to the margin wheat resource vegetation on aphid numbers for each genotype. (A) Chinese Spring genotype: only the developmental stage had an effect on the number of aphids found, with more aphid attacking mature plants. (B) Rotem genotype: more aphids were detected on advanced developmental stages and closer to the neighboring wheat resource. (C) Svevo genotype: more aphids were detected on advanced developmental stages and closer to the neighboring wheat resource. (D) Zavitan genotype: a significant difference was detected in the plant position relative to marginal wheat resource.

Additionally, we evaluated the effects of phenology and distance from the marginal wheat resource vegetation on aphid abundance regardless of the wheat genotype. The model presents significant effects for plant phenology (*F*_1,531_ = 85.04, *P* value < 0.001) and position (*F*_1,531_ = 78.35, *P*-value < 0.001) as well for their interaction (*F*_1,531_ = 73.76, *P*-value < 0.001). As shown in Fig. 5, most plants were in the flowering stage (10.5 Feekes scale). Plants that were closer to the wheat resource vegetation (1–5; yellow and orange color) hosted more aphids than the distant ones (13–16; gray and black color). We further tested the effects of phenology and distance from wheat resource vegetation on aphid abundance for each wheat type separately (Fig. 6). In Chinese Spring, only phenology significantly affected the number of aphids (*F*_2,127_ = 11.18, *P*-value < 0.001), while distance (*F*_1,127_ = 0.01, *P*-value = 0.996), and the interaction (*F*_1,127_ = 0.01, *P*-value = 0.995) did not have significant effects. In Zavitan, only distance from the wheat resource vegetation affected aphid abundance (*F*_1,116_ = 5.16, *P*-value = 0.025), while phenology (*F*_2,116_ = 1.11, *P*-value = 0.331), and their interaction term (*F*_1,116_ = 1.83, *P*-value = 0.165) were not significant. Both Svevo and Rotem had more aphids on older developmental stages closer to the neighboring wheat field, as shown by the following values: Svevo phenology (*F*_2,141_ = 15.60, *P*-value < 0.001), distance (*F*_1,141_ = 5.23, *P*-value = 0.024), and interaction (*F*_1,141_ = 4.15, *P*-value = 0.018); and Rotem phenology (*F*_2,125_ = 42.52, *P* value < 0.001), distance (*F*_1,125_ = 66.36, *P*-value < 0.001), and interaction (*F*_1,125_ = 10.13, *P*-value = 0.002). The distance of focal wheat relative to the margin wheat resource vegetation and phenology relative to aphid abundance on the four wheat genotypes (combined data) is presented in Fig. S4. Overall, we found that wheat plants that were located closer to wheat resource vegetation were more developmentally advanced and hosted more aphids compared to the plants that were located further from the wheat resource, closer to the natural resource vegetation (Figs. 5 and 6).

## Discussion

Interspecific genetic diversity in plants is frequently studied to elucidate the genetic elements related to biotic and abiotic resistances (Chandrasekhar, Nashef & Ben-David, 2017; Avni et al., 2018; Shaar-Moshe et al., 2019). To better understand the effect of genetic factors on biotic stresses such as aphid infestation, we exploited natural wheat variety, represented here by wild emmer and domesticated *Triticum* genotypes. Our results indicate that the four selected genotypes varied in terms of aphid susceptibility and phenology (Figs. 1 and 2). The domesticated Rotem variety was the most aphid-susceptible genotype, whereas the wild emmer wheat, Zavitan, demonstrated superior resistance. It has previously been suggested that several agriculturally important traits were reduced as a result of domestication (Chaudhary, 2013). For example, decreased levels of resistance to herbivore attack (Luedders & Dickerson, 1977), bacterial blight (Ashfield et al., 1998), and fungal disease (Garcia et al., 2008) were documented in domesticated members of the Fabaceae family compared to their wild progenitors. Reduced genetic variation during selection for beneficial traits associated with crop production (Harlan, 1976) and co-evolution of insect herbivores under chemical pest control (Kennedy & Barbour, 1992) both likely contribute to increased susceptibility of crops to insect attack as a result of domestication (Sotherton & Emden, 1982). Furthermore, previous screenings of 27 barley (*Hordeum*) genotypes revealed that wild progenitors are more resistant to *R. padi* aphids compared to the cultivated genotypes (Weibull, 1987). On the other hand, a recent large-scale study spanning five decades of wheat breeding progress in western Europe suggested that the intense selection process improved wheat production under the influence of favorable as well as some non-favorable agrochemical inputs (Voss-Fels et al., 2019). Based on the selected wheat genotypes used in this study, we emphasize that crop domestication may increase aphid abundance in field growth conditions, which could be a result of efficiency of defense mechanisms.

Phenological variations in host plants are known to affect plant-insect interactions due to changes in the quality and quantity of food resources (Hunter, 1992; Van Asch & Visser, 2007). In this work, the hexaploid bread wheat Rotem genotype presented the fastest growth (most plants were at the advanced developmental stages of heading and flowering), while the other bread wheat genotype, Chinese Spring, demonstrated the slowest growth (most plants were in a stage of last leaf just visible, ligule of the last leaf visible, or booting; Fig. 2). Insects can change their feeding site preferences or reproduction rates according to the developmental stage of the host plant (Leather & Dixon, 1981). Therefore, plants can modify their phenology to delay growth or to escape herbivory by an early transition into the reproduction stage (Kessler & Halitschke, 2007; Mitchell et al., 2016). Plant–insect interaction also relies on the dynamic populations of herbivores and their natural enemies throughout the growing season (De Geyter et al., 2007; Amin et al., 2019). It was previously reported that temporal shifts might affect the synchronization of plant–pest life cycles (Médiène et al., 2011). Thus, early sown spring cereals might have enough time to complete the vulnerable seedling stage and avoid aphid infestation. In this work, we counted aphid numbers and scored wheat phenology twice during the growing season at an interval of ten days, the results of which showed significant differences (Figs. 1 and 2).

The four wheat genotypes showed significant differences in aphid abundance (Fig. 1), which was also affected by the position of the wheat plant (Fig. 6). The results indicate that the phenological plasticity depends on the combined effects of genotypic background and distance from marginal vegetation. The large number of aphids on the focal plants adjacent to the marginal wheat resource could be the result of low natural enemy recruitment (Chaplin-Kramer et al., 2011), which could result from variation in the plants’ volatile signals (Kessler & Halitschke, 2007; Kessler & Kalske, 2018). These plants are challenged by a higher abundance of aphids, which they might avoid by transitioning early into the reproduction stage (Mitchell et al., 2016).

The metabolic constituent of resistance to aphids is a combination of chemical defenses and adjustments to plant palatability and nutritional quality (Zhou et al., 2015). We had previously detected a massive metabolic variation between Svevo and Zavitan genotypes in response to *Rhopalosiphum maidis* (corn leaf aphid) infestation, including changes in phytohormone levels (Chandrasekhar et al., 2018). However, these experiments were conducted during the seedling stage (2-weeks old) and under controlled growth conditions. Young wheat plants synthesize benzoxazinoids as a defense against insects (Sue, Nakamura & Nomura, 2011; Makowska, Bakera & Rakoczy-Trojanowska, 2015; Batyrshina et al., 2020), but the compounds were not detected in later developmental stages (either juvenile or mature), except for in the dry seeds (Hanhineva et al., 2011). In the metabolic analysis in the present study, we identified three flavonoids, namely 3-caffeoylquinic acid (also known as chlorogenic acid), quercetin-3-O-rutinoside (also known as rutin), and kaempferol-3-glucoside. The levels of these were significantly different between the four genotypes and between the two sampling times (Fig. 4). Flavonoids are widely distributed specialized metabolites with multiple metabolic functions in plants, including defense against UV-B radiation, pest infestation, and pathogen infection, as well as enhancing rhizobacteria nodulation and pollen fertility (Cornell & Hawkins, 2003). Biosynthesis of 3-caffeoylquinic acid is known to be induced by insect herbivory and plays a defensive role in many plant species, including tomato (*Solanum lycopersicum*), potato (*Solanum tuberosum*) and others (Leiss et al., 2009; Kundu, Vadassery & Pineda, 2019). Quercetin-3-O-rutinoside and kaempferol-3-glucoside (Tohge, De Souza & Fernie, 2017) are known to possess various functions, including filtering UV radiation and as components of interactions with other organisms, such as microbes, insects and neighboring plants (Zhang, Zhao & Qiu, 2013). Our results suggest that flavonoids are present in varying levels between the four wheat genotypes and are reduced over time. However, there was not a clear relationship between aphid abundance and the levels of these compounds. The level of sucrose was significantly reduced, while the amino acids Val and Trp were significantly increased between the first and second sampling time points (Fig. 4). These changes in central metabolites also influence insect feeding (Zhou et al., 2015); however, this requires further investigation.

The resource concentration hypothesis predicts that specialist insect herbivores are more abundant (density per unit of the host-plant species) when their host plants grow in high-density patches and low-diversity mixtures (Altieri, 1995; Rhainds & English-Loeb, 2003). This hypothesis arose from studying insect pests in agricultural crop systems. By contrast, other studies have described the specialist insect herbivore abundance of natural or seminatural communities and the “resource dilution” effect on monocultures (Otway, Hector & Lawton, 2005). In this case, the impact of neighboring marginal plants on insect abundances on focal plants might result in associational resistance or higher susceptibility to the herbivores (Kos et al., 2015). In our study, we found that aphid abundance was higher on the focal plants located closer to the monoculture wheat resource, supporting a resource concentration effect. However, it is still unknown whether this is related to an abundant presence of natural enemies.

## Conclusions

We propose that spatial position plays an important role in the determination of plant resistance to pests, in addition to the crucial effect of wheat genotype and, subsequently, wheat phenology. Under pest pressure, farmers routinely utilize synthetic pesticides to mitigate yield loss (Popp, Pető & Nagy, 2013). This approach has some drawbacks, such as increased pesticide resistance in target herbivores and the collapse of beneficial insect populations such as pollinators or natural enemies. To improve pest management, additional information on the combined influence of crop genetic background and spatial position of individual plants could improve predictive modeling of insect abundance and distribution. Overall, this study highlights the importance of margin vegetation as a part of the agroecosystem that may be further exploited for effective, sustainable practices for reducing biotic stresses and pesticide usage.

## Supplemental Information

10.7717/peerj.9142/supp-1 Supplemental Information 1 Tables S1-S4. Table S1: Raw data used in this study.
Table S2: Classification of natural vegetation grown the west side of the field.
Table S3: Untargeted metabolic raw data generated by the UPLC-qTOF-MS in negative and positive ion modes.
Table S4: List of metabolites identified in leaf tissues of 3-month-old wheat plants by UPLC-qTOF-MS analysis.

10.7717/peerj.9142/supp-2 Supplemental Information 2 Figures S1-S4. Figure S1: A field setup. A) A schematic illustration of the three blocks and the division into four genotypes. B) A photo of the filed. C) A schematic illustration of the field setup.
Figure S2: Aphid abundance in the experimental blocks. Number of aphids from the focal wheat plants were counted twice: on March 20th and April 1st, 2018 (mean ± SE, number of replicates Block1-92; Block2-88, and Block3-87). No significant differences were found within blocks for same counting date, using the one-way ANOVA (Tukey-Kramer HSD post-hoc tests).
Figure S3: A scatter plot between the average of phenology and aphid performance of the four wheat genotypes in two counting dates. CS, Chinese Spring, RT, Rotem, SV, Svevo, ZT, Zavitan. Counting #1, on March 20th, 2018 and counting #2 on April 1st, 2018. The developmental stages presented as continues values. The Pearson correlation coefficient r value = 0.64.
Figure S4: Effect of phenology and position in relation to the margin wheat resource vegetation on aphid numbers counted on the four wheat genotypes (combined data).

## Additional Information and Declarations

### Data Availability

The following information was supplied regarding data availability: Raw data tables are available in the Supplemental Files.
