# Supplementary material for: The combined impacts of wheat spatial position and phenology on cereal aphid abundance
Source: PeerJ. 2020 May 26;8:e9142. doi: 10.7717/peerj.9142 (PMC7258891; doi:10.7717/peerj.9142)
Supplement: Supplemental Information 2 — Figure S1: A field setup. A) A schematic illustration of the three blocks and the division into four genotypes. B) A photo of the filed. C) A schematic illustration of the field setup. Figure S2: Aphid abundance in the experimental blocks. Number of aphids from the focal wheat plants were counted twice: on March 20th and April 1st, 2018 (mean ± SE, number of replicates Block1-92; Block2-88, and Block3-87). No significant differences were found within blocks for same counting date, using the one-way ANOVA (Tukey-Kramer HSD post-hoc tests). Figure S3: A scatter plot between the average of phenology and aphid performance of the four wheat genotypes in two counting dates. CS, Chinese Spring, RT, Rotem, SV, Svevo, ZT, Zavitan. Counting #1, on March 20th, 2018 and counting #2 on April 1st, 2018. The developmental stages presented as continues values. The Pearson correlation coefficient r value = 0.64. Figure S4: Effect of phenology and position in relation to the margin wheat resource vegetation on aphid numbers counted on the four wheat genotypes (combined data). [file peerj-08-9142-s002.pptx]

## Slide 1
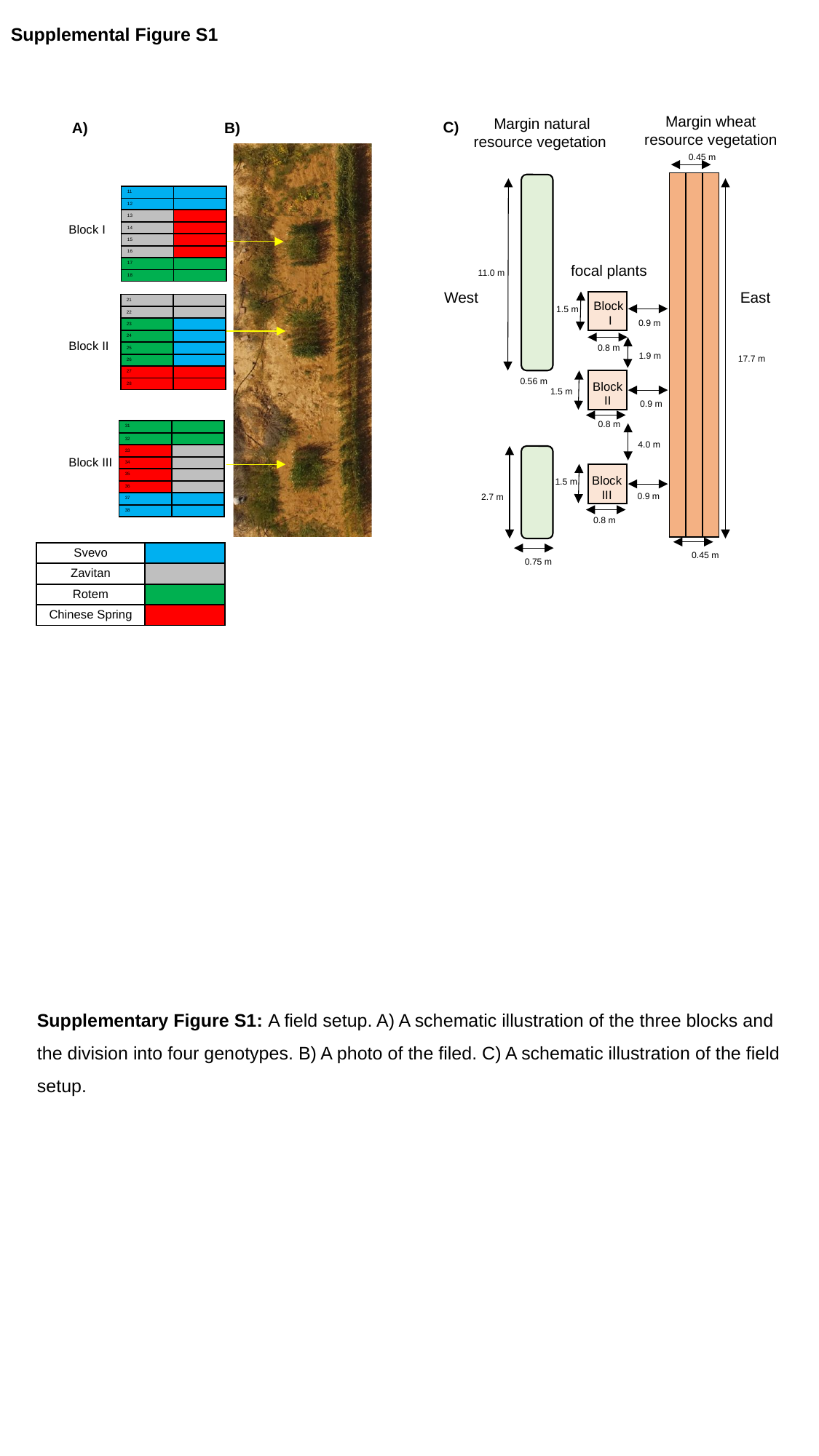

Supplemental Figure S1
Margin wheat
resource vegetation
Margin natural resource vegetation
C)
A)
B)
0.45 m
| | | |
| --- | --- | --- |
| 11 | |
| --- | --- |
| 12 | |
| 13 | |
| 14 | |
| 15 | |
| 16 | |
| 17 | |
| 18 | |
Block I
Block II
Block III
focal plants
11.0 m
East
West
Block
 I
| 21 | |
| --- | --- |
| 22 | |
| 23 | |
| 24 | |
| 25 | |
| 26 | |
| 27 | |
| 28 | |
1.5 m
0.9 m
0.8 m
1.9 m
17.7 m
0.56 m
Block
II
1.5 m
0.9 m
0.8 m
| 31 | |
| --- | --- |
| 32 | |
| 33 | |
| 34 | |
| 35 | |
| 36 | |
| 37 | |
| 38 | |
4.0 m
Block
III
1.5 m
0.9 m
2.7 m
0.8 m
| Svevo | |
| --- | --- |
| Zavitan | |
| Rotem | |
| Chinese Spring | |
0.45 m
0.75 m
Supplementary Figure S1: A field setup. A) A schematic illustration of the three blocks and the division into four genotypes. B) A photo of the filed. C) A schematic illustration of the field setup.

## Slide 2
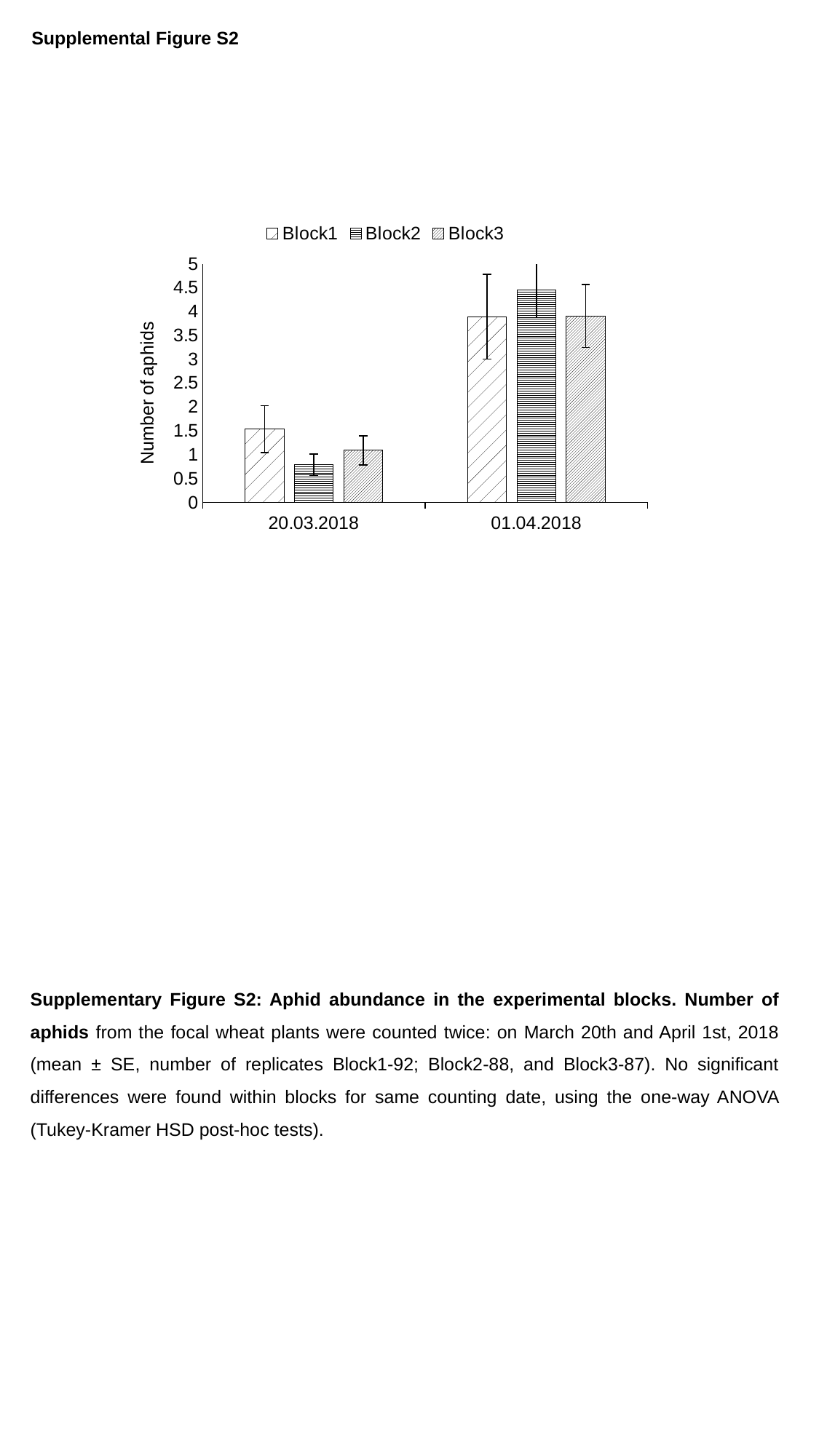

Supplemental Figure S2
### Chart
| Category | Block1 | Block2 | Block3 |
|---|---|---|---|
| 20.03.2018 | 1.5326086956521738 | 0.7954545454545454 | 1.0919540229885059 |
| 01.04.2018 | 3.891304347826087 | 4.454545454545454 | 3.9080459770114944 |Supplementary Figure S2: Aphid abundance in the experimental blocks. Number of aphids from the focal wheat plants were counted twice: on March 20th and April 1st, 2018 (mean ± SE, number of replicates Block1-92; Block2-88, and Block3-87). No significant differences were found within blocks for same counting date, using the one-way ANOVA (Tukey-Kramer HSD post-hoc tests).

## Slide 3
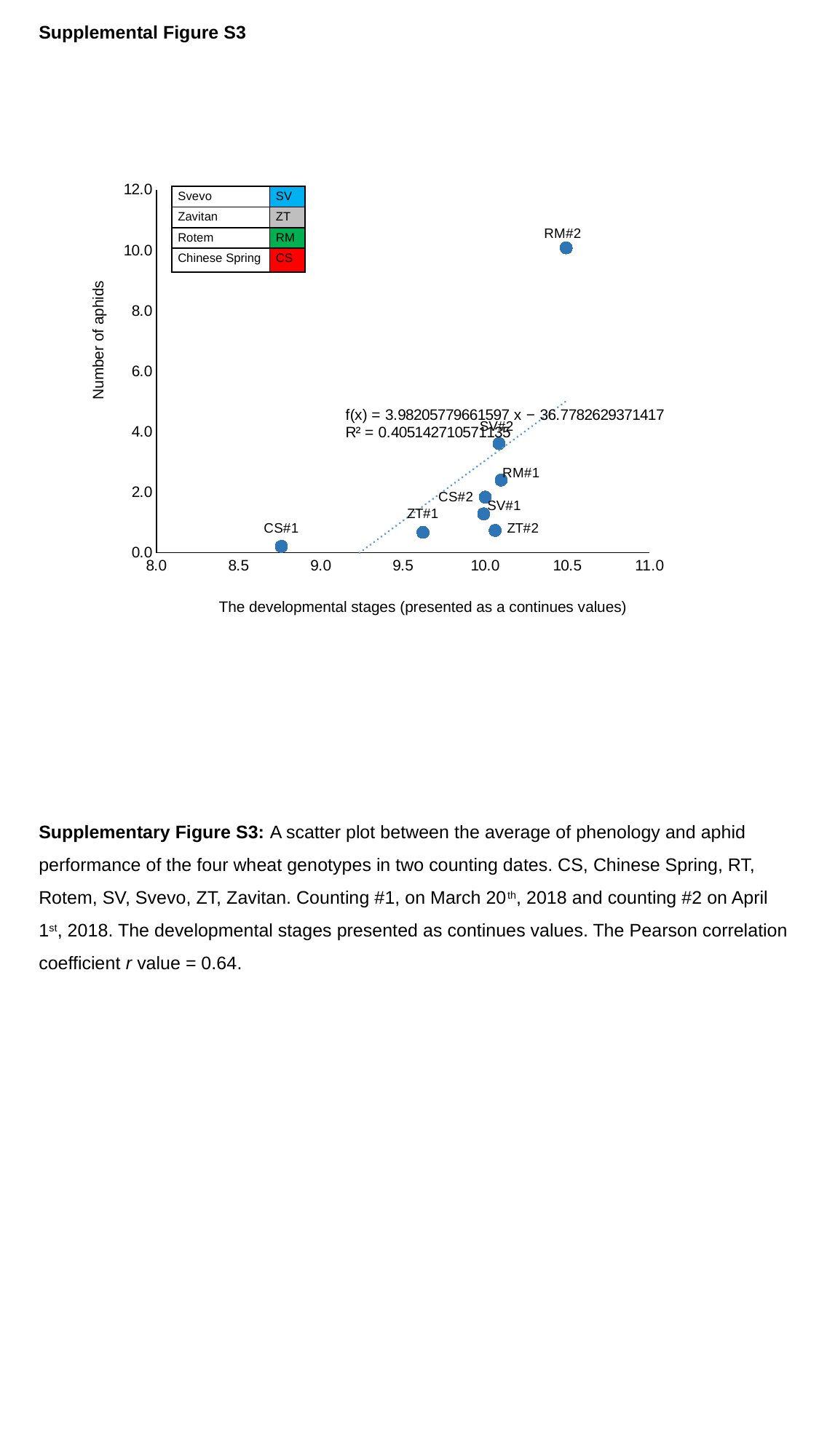

Supplemental Figure S3
### Chart
| Category | |
|---|---|| Svevo | SV |
| --- | --- |
| Zavitan | ZT |
| Rotem | RM |
| Chinese Spring | CS |
Number of aphids
The developmental stages (presented as a continues values)
Supplementary Figure S3: A scatter plot between the average of phenology and aphid performance of the four wheat genotypes in two counting dates. CS, Chinese Spring, RT, Rotem, SV, Svevo, ZT, Zavitan. Counting #1, on March 20th, 2018 and counting #2 on April 1st, 2018. The developmental stages presented as continues values. The Pearson correlation coefficient r value = 0.64.

## Slide 4
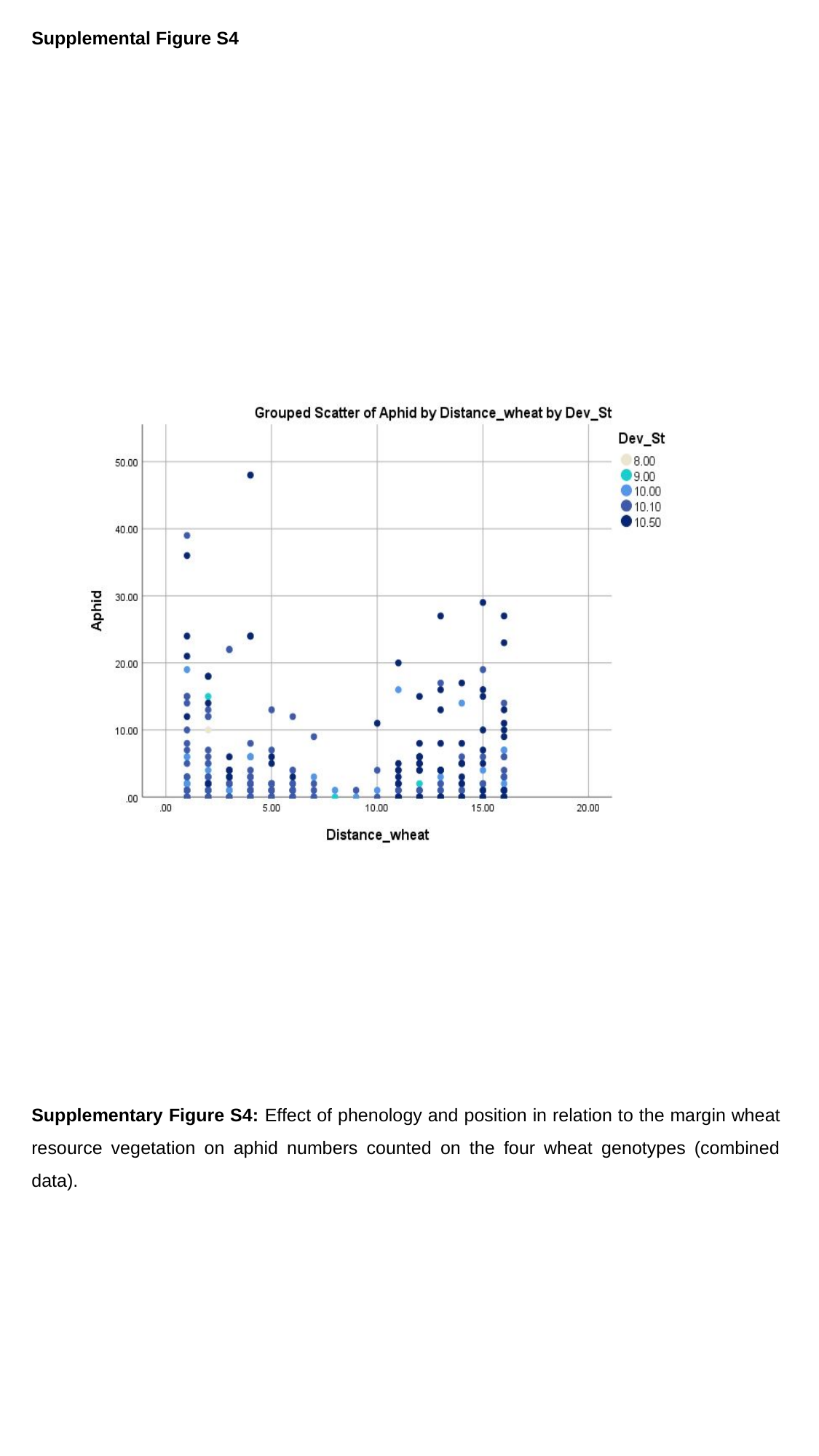

Supplemental Figure S4
Supplementary Figure S4: Effect of phenology and position in relation to the margin wheat resource vegetation on aphid numbers counted on the four wheat genotypes (combined data).
